# Exploring the relationship between reproductive aging, arterial stiffness, and cardiorespiratory fitness in women after menopause

**DOI:** 10.14814/phy2.70722

**Published:** 2026-03-29

**Authors:** Luís Ferreira, Catarina Abrantes, Maria Emília Alves, Lucimere Bohn, Helena Moreira

**Affiliations:** ^1^ Department of Sports Science Douro Higher Institute of Educational Sciences, CI‐ISCE Penafiel Portugal; ^2^ Department of Sports Science, Exercise and Health University of Trás‐os‐Montes and Alto Douro Vila Real Portugal; ^3^ Research Center in Sports Sciences, Health Sciences and Human Development (CIDESD) University of Trás‐os‐Montes and Alto Douro Vila Real Portugal; ^4^ Research Centre in Physical Activity, Health, and Leisure (CIAFEL) Faculty of Sport at the University of Porto Porto Portugal; ^5^ Faculty of Sport at the University of Porto Porto Portugal; ^6^ Research Centre in Sport, Physical Education, Exercise and Health (CIDEFES) Porto University Center Porto Portugal; ^7^ Centre for the Research and Technology of Agro‐Environmental and Biological Sciences‐CITAB, Inov4Agro University of Trás‐os‐Montes and Alto Douro, UTAD Quinta de Prados Vila Real Portugal

**Keywords:** aging, cardiovascular, fitness, hemodynamics, postmenopause

## Abstract

This cross‐sectional study examined associations between postmenopausal stage (early and late), arterial stiffness, and cardiorespiratory fitness. A total of 125 postmenopausal women (58.07 ± 6.61 years) underwent carotid‐femoral pulse wave velocity assessment (SphygmoCor, Atcor). A subset of 96 women (57.20 ± 6.42 years) completed an estimated maximal oxygen uptake testing, using a Monark Ergomedic 839E cycle ergometer. Multiple stepwise regressions identified predictors of arterial stiffness and cardiorespiratory fitness. Compared with late postmenopause, early postmenopausal women showed lower arterial stiffness (−0.71 ± 0.29 m/s, *p* = 0.02) and systolic blood pressure (−6.40 ± 3.06 mmHg, *p* = 0.04), indicating a relative difference within postmenopause. Early postmenopause also showed higher cardiorespiratory fitness levels than late postmenopause. Moreover, body fat percentage was inversely associated with cardiorespiratory fitness in +1c substage, and visceral fat level displayed inverse associations with cardiorespiratory fitness in late postmenopause. In the +1a substage, hormone therapy was positively associated with cardiorespiratory fitness in exploratory model. Within postmenopause, early postmenopause showed lower arterial stiffness and systolic blood pressure than late postmenopause, and blood pressure and adiposity emerged as key correlates of vascular stiffness and fitness. While causal inference is precluded by the cross‐sectional design, the results support stage‐targeted strategies emphasizing BP control, body‐composition management, and exercise to help preserve vascular and functional health in women after menopause.

## Graphical abstract

Arterial stiffness and cardiorespiratory fitness across postmenopause.

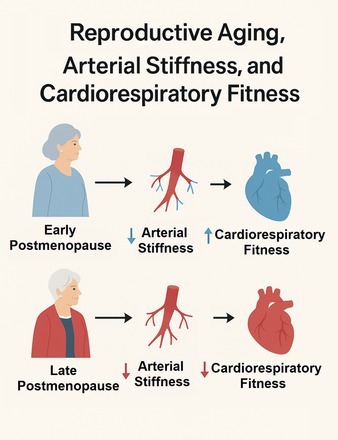

## 1 INTRODUCTION

Cardiovascular disease (CVD) incidence and mortality are closely associated with aging and prolonged exposure to modifiable risk factors (Cao et al., 2020; Ferreira et al., 2024; Kim & Choi, 2023). Among women, the menopausal transition represents a critical period marked by rapid physiological changes, including accelerated vascular aging (Hildreth et al., 2014; Nowak et al., 2018). Menopause also exacerbates the risk of CVD (Cao et al., 2020; Stamatelopoulos et al., 2020), due to various factors, including a decline in estrogen levels which leads to adverse changes in lipid profiles and elevated blood pressure, fostering atherosclerosis (Shufelt & Manson, 2021). Scientific literature indicates that postmenopausal women experience increases in carotid intima‐media thickness and in the adventitial diameter, leading to elevated arterial stiffness (AS) (El Khoudary et al., 2020; Samargandy et al., 2020). These wall changes begin in the menopausal transition and extend into the first year after the final menstrual period, with a more pronounced effect observed in the early postmenopause stage (El Khoudary et al., 2020). Consistent cross‐sectional data also indicate that menopause independently augments the age‐related increase in AS (Zaydun et al., 2006).

Beyond body‐composition shifts (e.g., increased central adiposity), menopausal women frequently develop elevated blood pressure and a higher prevalence of metabolic syndrome (Solanki et al., 2021; Tikhonoff et al., 2019), factors mechanistically linked to AS and adverse cardiovascular outcomes (El Khoudary et al., 2020; Karagkouni et al., 2022). Lifestyle changes around this period, including reductions in habitual physical activity (Ko & Jung, 2021), further interact with hemodynamic and metabolic profiles (Rossman et al., 2018; Santos‐Parker et al., 2014).

Despite advances, important knowledge gaps remain. Prior work has established that vascular dysfunction and large‐artery stiffening worsen from pre‐ to peri‐ and postmenopause (e.g., impaired endothelial function and increased central AS) (El Khoudary et al., 2020; Hildreth et al., 2014; Samargandy et al., 2020). What is less clear is the extent to which the specific phenotypes under study, AS and cardiorespiratory fitness (CRF), differ between early (EP) versus late postmenopause (LP) and how they relate to age, blood pressure, and adiposity within EP substages. Importantly, our design compares EP and LP and evaluates associations within EP substages (+1a, +1b, and +1c), rather than including a premenopausal control group. Therefore, observed differences are interpreted as relative contrasts within postmenopause.

Accordingly, we aimed to describe how age, body composition, blood pressure, and menopausal characteristics relate to AS (carotid–femoral pulse wave velocity, cfPWV) and CRF (estimated VO_2max_) across EP and LP stages and within EP substages. This approach evaluates clinically relevant correlates of vascular and functional phenotypes in postmenopausal women and refines hypotheses for future longitudinal work.

## 2 MATERIALS AND METHODS

This is a cross‐sectional study and is part of the research project Meno(s)Pausa+Movimento (Moreira et al., 2024), an exercise‐based program with community intervention hosted by the Municipality of Penafiel, Portugal, and with the scientific and pedagogical coordination of the University of Trás‐os‐Montes and Alto Douro.

Data was collected between February 22 and April 9, 2022. Participants were reached through personal invitations, social media, and information disseminated at medical appointments in the city's primary care centers and local newspapers.

### 2.1 Participants

The study involved 125 postmenopausal women, in early postmenopause (EP, up to 6 years after menopause) and late postmenopause (LP, more than 6 years after menopause).

Of the 125 study participants (mean age = 58.07 ± 6.61 years; 58 in the EP group and 67 in the LP group), valid CRF tests and data were available for 96 postmenopausal women (mean age = 57.20 ± 6.42 years; 48 in the EP group and 48 in the LP group). The CRF assessment test was interrupted in the following situations: when participants were unable to complete at least two levels of the protocol (*n* = 15) due to local fatigue; upon reaching 85% of their maximum heart rate for safety (*n* = 5); due to orthopedic pain that prevented continuation (*n* = 6); due to heart rate monitor failures that impeded recording (*n* = 2); or as a result of medication changes (*n* = 1).

Inclusion in the study was preceded by an assessment of clinical and reproductive history by a physician and applied the following criteria: (1) postmenopausal women (Harlow et al., 2012); (2) be aged 42 years or older; (3) have no history of premature ovarian insufficiency (defined as menopause before age 40); (4) have no history of angina or myocardial infarction symptoms in the past 3 months; (5) not be taking beta‐blockers or anti‐arrhythmic medications; and (6) have no musculoskeletal limitations that would hinder physical performance assessments. The study included women with natural menopause (defined as 12 consecutive months of amenorrhea not attributable to any identifiable pathological or physiological cause) as well as women with induced menopause, resulting from medical depletion of the ovarian follicular reserve.

The number of term pregnancies, as well as medication influencing blood pressure (BP) and hormonal therapy, were considered in the statistical analyses as potential influencing variables. The length of estrogen exposure was also considered and determined by measuring the time from the onset of menarche (first menstrual period) to the onset of menopause (last menstrual period). This timeframe represents the total years of natural estrogen production, which is an important factor for assessing its cumulative relationship to health outcomes in postmenopausal women.

To classify menopausal stages, the month and year of each participant's last menstrual period were recorded. Using this data and the date of data collection, the number of years since the onset of amenorrhea was calculated. This classification enabled the subdivision of early postmenopause (EP) into stages +1a, +1b, and +1c, and late postmenopause (LP; stage +2), according to the criteria established by Harlow et al. (2012).

This rigorous methodology ensured precise group formation, minimizing misclassification, and enhancing the validity of comparisons between postmenopause stages.

### 2.2 Measures

#### 2.2.1 Anthropometry/body composition

Height (m) was assessed using a SECA 220 stadiometer (Seca Corporation, Hamburg Germany). Participants were barefoot, and repeated results were recorded after a deep inspiration (Heyward & Wagner, 2004), considering a tolerance limit of 2 mm (Sobral, 1985). Body mass (kg), fat mass (%), and visceral fat level (VFL) (points) were assessed using InBody 120 octopolar bioimpedance (Biospace, Seoul, Korea), following the recommended procedures set out in the literature (Biospace, 2021; Heyward & Wagner, 2004). The cutoff points for elevated central adiposity and obesity were, respectively: VFL > 9 points (Biospace, 2021) and fat mass ≥ 35% in women (Lohman et al., 1997).

#### 2.2.2 Hemodynamics

Blood pressure (BP) and AS measurements were conducted by a single well‐trained researcher. Participants rested in a supine position on an observation table for 10 min in a quiet, temperature‐controlled room (24.7°C ± 0.66°C) with 50.2% (±5.12) humidity (TensioMed, 2012; Van Bortel et al., 2012). An automatic BP monitor OMRON 705IT (HEM‐759‐E, Omron Corporation, Kyoto, Japan) (Coleman et al., 2006) was used with their left arm resting at heart level (Van Bortel et al., 2012). The cuff was wrapped around the brachial artery, with the size of the cuff being adapted to arm circumference to ensure the accuracy of the measurement (medium 22–32 cm; large 32–42 cm) (Magyari et al., 2018). At least two measurements were taken (>1 min apart), and additional measurements were made when differences between the two first were superior to 2 mmHg (Magyari et al., 2018). The mean value between the 2 closest measurements was recorded as the final systolic (SBP) and diastolic blood pressure (DBP) (Magyari et al., 2018).

To ensure accurate readings, participants were instructed to fast for over 4 h, refrain from smoking, alcohol, and caffeine consumption for 8 h, and avoid physical exercise for 24 h before the assessments. Talking and sleeping were not allowed during the entire procedure (Van Bortel et al., 2012).

Vascular stiffness was assessed by measuring carotid‐femoral pulse wave velocity (cfPWV), using applanation tonometry (SphygmoCor, AtCor Medical, Australia) and following international guidelines (Van Bortel et al., 2012).

Consecutive pressure waves were recorded at the right carotid and femoral arteries, accompanied by simultaneous electrocardiogram (ECG) recordings, with data being registered during at least two respiratory cycles (about 10–12 s) (Bohn et al., 2020). The R‐wave of the ECG provided a reference for determining the transit time of the wave between the two recording sites (using the foot‐to‐foot method). The distance traveled by the pressure wave was calculated as the direct distance between the femoral and carotid recording points, adjusted by a factor of 0.8 (Bohn et al., 2020).

The cfPWV value was determined by dividing the direct distance (measured in meters) by the transit time (measured in seconds). The same trained researcher conducted each measurement twice. Measurements were accepted as valid if the difference between attempts was equal to or less than 0.5 m/s (Kollias et al., 2020; Van Bortel et al., 2012). If the discrepancy exceeded 0.5 m/s, a third measurement was obtained. In such instances, extreme results were discarded, and new measurements were taken until there was a gap between measurements of up to 0.5 m/s. The cut‐off points considered for elevated cfPWV were 10 m/s (Van Bortel et al., 2012).

#### 2.2.3 Cardiorespiratory fitness

Cardiorespiratory fitness was assessed indirectly by estimating VO_2max_, using the YMCA submaximal protocol (Zhang et al., 2017) on a cycle ergometer (Monark Ergomedic mod. 839E, Monark, Vansbro, Sweden) calibrated every half‐day of use.

The test typically involves at least two, and possibly three, exercise stages, each lasting 3 min, resulting in a total test duration of 6–9 min (Zhang et al., 2017). Participants were required to complete two separate workloads, resulting in HR values between 110 and 150 beats per minute (bpm) (Kaminsky, 2010). All participants started with a workload of 150 kg·m·min^−1^ in the first stage, and the workload for the second stage (between 300 and 750 kg·m·min^−1^) was determined by the HR recorded during the first stage (Kaminsky, 2010). A third stage was performed when the HR of the second stage was lower than 110 bpm. In such cases, the third workload was raised incrementally by 150 kg·m·min^−1^ to ensure an appropriate submaximal HR response. It was imperative that the participant maintained a pedal rate of 50 (±2) revolutions per minute throughout the test (Beekley et al., 2004; Kaminsky, 2010; Zhang et al., 2017), assisted by a well‐trained researcher and a metronome.

Age‐based cutoff values were used to assess the participants' CRF levels and their relationship on cardiovascular health (Pescatello, 2014). A VO_2max_ ≥ 29 mL.kg^−1^.min^−1^ was considered normal for participants aged 46–55 years, ≥26 mL.kg^−1^.min^−1^ for those aged 56–65 years, and ≥23 mL.kg^−1^.min^−1^ for those over 65 years.

### 2.3 Statistical methods

The statistical analysis was conducted using IBM SPSS Statistics for Windows version 29.0 (IBM Corp, Armonk, NY, USA), with a significance level set at *p* ≤ 0.05. Continuous variables were presented as mean ± standard deviation, while categorical variables were summarized as frequencies and percentages.

For most variables, the two groups of postmenopausal women were compared using the Student's *t*‐test for independent samples. Normality within the EP and LP groups was assumed based on the sample size, as each group included more than 30 participants. According to the central limit theorem, sufficiently large sample sizes (*n* > 30) ensure that the sampling distribution of the mean approximates a normal distribution (Kwak & Kim, 2017).

An analysis of covariance (ANCOVA) was employed to compare AS and CRF between the two main groups, while controlling for potential confounding variables, specifically age and BP. Although hormone therapy was initially examined, it was not included as a covariant in the overall ANCOVA model due to the lack of statistically significant influence at the global group level. Additionally, Cohen's d effect size was calculated to evaluate the magnitude of the observed differences. Also, for blood pressure comparisons, we first assessed ANCOVA assumptions. The homogeneity of regression slopes (stage × age) was tested and found to be violated, indicating nonparallel age–BP relationships across groups. Therefore, ANCOVA‐adjusted means were deemed inappropriate and were not reported.

Comparisons between the EP subgroups were performed using a one‐way ANOVA (Table 3). The Shapiro–Wilk test was used to assess normality, with age, %BF, VFL, SBP, and DBP showing normal distributions, while the number of term pregnancies, time of estrogen exposure, and cfPWV did not. Logarithmic (Log10), square root, and quadratic transformations were tested; only cfPWV achieved normality after log transformation and was included in the ANOVA. Homogeneity of variances was confirmed with Levene's test (*p* = 1.52), and Bonferroni post hoc was applied for pairwise comparisons.

Multiple stepwise regression analyses were conducted to identify significant variables of AS and CRF. The independent variables included age, number of term pregnancies, duration of estrogen exposure, time since menopause, fat mass, VFL, SBP, and DBP. Multicollinearity was assessed based on the correlation degree of the independent variables (r), the proportion of their variation explained by the other variables included in the model (tol), the variance inflation factor (VIF = 1/tol), the condition index (CI), and the proportion of variance for each of the estimated regression coefficients (PV). Models were rejected if CI > 30, *r* < 0.90, tol > 0.10, VIF < 10, or PV < 90% for more than one coefficient (Pestana & Gageiro, 1998).

## 3 RESULTS

Among all participants, 114 (91.2%) postmenopausal women underwent natural menopause. Hormone therapy was reported by 25 women (20%), and 87 (69.6%) indicated they were not taking any BP lowering medication. Additionally, 96 women (76.8%) were classified as obese, and 102 (81.6%) presented central obesity (Table 1).

**TABLE 1:** Distribution of demographic and clinical factors in relation to arterial stiffness and cardiorespiratory fitness.

Variables		Arterial stiffness sample	Cardiorespiratory fitness sample
		(*n* = 125)	(*n* = 96)
		*n* (%)	*n* (%)
Nature of menopause	Natural	114 (91.2)	87 (90.6)
	Induced	11 (8.8)	9 (9.4)
Hormone therapy	No	100 (80.0)	77 (80.2)
	Yes	25 (20.0)	19 (19.8)
Medications that affect blood pressure	No	87 (69.6)	68 (70.8)
	Yes	38 (30.4)	28 (29.2)
Full‐term pregnancies	0	10 (8.0)	4 (4.2)
	1–2	90 (72.0)	73 (76.0)
	3–4	23 (18.4)	17 (17.7)
	5	2 (1.6)	2 (2.1)
Obesity	No	29 (23.2)	21 (21.9)
	Yes	96 (76.8)	75 (78.1)
Central obesity	No	23 (18.4)	16 (16.7)
	Yes	102 (81.6)	80 (83.3)

In addition, the study included an analysis of CRF within a subset of the participants. Of the 125 postmenopausal participants, 96 had valid VO_2max_ assessments and were included in the CRF analyses. Of these, 90.6% had experienced natural menopause, 80.2% were not using hormone therapy, and 70.8% were not taking any medication that influenced blood pressure.

In the AS cohort (*n* = 125), 69.6% (87) were not taking medications that affect blood pressure, and within its CRF subsample (*n* = 96), this corresponded to 79.8% (68) of the participants. Most women had one to two full‐term pregnancies (representing 72% in the AS sample and 76% in the CRF sample), and a smaller percentage had three to four pregnancies (representing 18.4% in the AS sample and 17.7% in the CRF sample).

Obesity was common in both analyses, with 76.8% of the AS sample and 78.1% of the CRF sample classified as obese. Central obesity was even more prevalent, affecting 81.6% of the AS sample and 83.3% of the CRF sample.

Considering the 125 women in the total sample (Table 2), the mean values of SBP and DBP were 131.34 ± 17.28 mmHg and 84.0 ± 8.54 mmHg, respectively. Compared to women in LP, those with a more recent menopause were younger (−8.75 ± 0.89 years, *p* < 0.01), had higher DBP values (3.92 ± 1.50 mmHg, *p* = 0.01), and lower SBP (6.40 ± 3.06 mmHg, *p* = 0.04) and cfPWV (−0.71 ± 0.29 m/s, *p* = 0.02).

**TABLE 2:** Comparison of physiological and clinical variables between women in early and late postmenopause in total sample reporting arterial stiffness (*n* = 125).

Variables	Total sample	Early postmenopause	Late postmenopause	Difference	*p*	Effect size (d)
	(*n* = 125)	Mean ± SD	Mean ± SD			
	Mean ± SD	(*n* = 58)	(*n* = 67)	Mean ± SD		
Age (years)	58.07 ± 6.61	53.38 ± 4.77	62.13 ± 5.13	−8.75 ± 0.89	<0.01*	−1.76
Full‐term pregnancies (*n*°)	1.89 ± 0.98	1.83 ± 0.94	1.94 ± 1.01	−0.11 ± 0.18	0.52	−0.12
Duration of estrogen exposure (years)	37.38 ± 4.18	38.10 ± 4.00	36.76 ± 4.10	1.35 ± 0.74	0.07	0.33
Body mass (kg)	69.92 ± 11.74	73.00 ± 12.68	67.26 ± 10.23	5.75 ± 2.05	0.01*	0.50
Height (m)	1.58 ± 0.06	1.59 ± 0.05	1.56 ± 0.06	0.03 ± 0.01	0.01*	0.45
Fat mass (%)	39.97 ± 6.86	40.54 ± 6.92	39.48 ± 6.82	1.06 ± 1.23	0.39	0.15
Visceral fat level (points)	13.76 ± 4.42	14.47 ± 4.59	13.15 ± 4.20	1.32 ± 0.79	0.10	0.30
Systolic blood pressure (mmHg)	131.34 ± 17.28	127.91 ± 17.90	134.31 ± 16.27	−6.40 ± 3.06	0.04* ^,^ ^a^	−0.38
Diastolic blood pressure (mmHg)	84.0 ± 8.54	86.1 ± 8.85	82.18 ± 7.87	3.92 ± 1.50	0.01*	0.47
Carotid‐femoral Pulse Wave Velocity (m/s)	8.16 ± 1.64	7.78 ± 1.58	8.48 ± 1.64	−0.71 ± 0.29	0.02* ^,^ ^b^	−0.44

*Note*: *p*, statistical significance; Effect size (d), standardized difference between the means of the subgroups, measured using Cohen's d.

Abbreviation: SD, standard deviation.

^a^ Statistical analysis was performed using the ANCOVA test, controlling for age.

^b^ Statistical analysis was performed using the ANCOVA test, controlling for age and blood pressure.

* *p* < 0.05.

Regarding body composition, the EP group had (*p* = 0.01) higher body mass (5.75 ± 2.05 kg) and greater height (0.03 ± 0.01 m). In both EP and LP women, the mean cfPWV values were below the clinical cutoff of 10 m/s.

Additionally, the analysis showed an overall prevalence of 15% for cfPWV >10 m/s, with 4% of cases in the EP group and 11% in the LP group. Due to this small and unbalanced distribution, no statistical comparison of proportions between EP and LP groups was performed.

Furthermore, Table 3 presents the mean values of the variables analyzed across the different substages of early postmenopause. A statistically significant difference in mean age was found between groups +1a and +1c (*p* < 0.01), with an expected progressive increase in age observed across the three substages.

**TABLE 3:** Comparison of physiological and clinical variables across early postmenopausal groups: Descriptive and ANOVA results (*n* = 58).

Variables	Early postmenopause (EP)			*F*	*p*	Tendency
	Mean ± SD					
	+1a (*n* = 17)	+1b (*n* = 15)	+1c (*n* = 26)			
						Post hoc
Age (years)	50.61 ± 4.06	52.16 ± 4.26	55.89 ± 4.31	8.44	<0.01	↑ a (*p* < 0.01)
Full‐term pregnancies (*n*)	1.65 ± 0.99	1.73 ± 0.88	2.00 ± 0.94	0.83	0.44	–
Duration of estrogen exposure (years) **#**	36.91 ± 4.22	37.89 ± 4.71	39.01 ± 3.81	1.21	0.31	–
Fat mass (%)	41.51 ± 6.35	39.99 ± 7.16	40.22 ± 7.33	0.28	0.76	–
Visceral fat level (points)	15.12 ± 4.41	11.33 ± 4.72	14.12 ± 4.76	0.34	0.72	–
Systolic blood pressure (mmHg)	130.71 ± 21.33	121.33 ± 16.11	129.89 ± 16.09	1.41	0.25	–
Diastolic blood pressure (mmHg)	87.71 ± 9.09	83.80 ± 8.77	86.39 ± 8.82	0.82	0.44	–
Carotid‐femoral Pulse Wave Velocity (m/s) **#**	7.71 ± 2.02	7.27 + 0.65	8.12 ± 1.59	1.41	0.25	–

*Note*: a, Significant differences between +1a and +1c; #, Logarithmic transformation of the distribution for normalization; **↑**, Upward tendency.

Also, parity (full‐term pregnancies) did not differ across EP subgroups (+1a, +1b, and +1c: *F* = 0.83, *p* = 0.44). Regarding body composition, hemodynamic parameters, and arterial stiffness, no statistically significant differences were found between the three groups.

Table 4 presents the stepwise regression models developed for AS. The data revealed a significant association of SBP on the variation of AS, particularly in stage +1b (*β* = 0.639, *p* = 0.01), where this variable independently accounted for 36.2% of the variation in cfPWV. In the EP group, higher SBP values (*β* = 0.451; *p* < 0.01) and time in menopause (*β* = 0.249; *p* < 0.01) explained cfPWV variation. In substage +1a, this variation is explained by VFL (*β* = 0.513; *p* = 0.01) and DBP (*β* = 0.445; *p* = 0.03). In substage +1b, SBP (*β* = 0.639) is the only predictor (*p* = 0.01). In substage +1c, SBP (*β* = 0.0.525) and time in menopause (*β* = 0.369) appeared again as significant predictors (*p* < 0.01 and *p* = 0.03, respectively). In the LP group, both age and SBP emerged as significant variables (*p* = 0.02) of cfPWV, with a standardized coefficient of 0.274 for both variables.

**TABLE 4:** Multiple regression analyses of arterial stiffness at distinct stages of menopause.

Arterial stiffness	β	*p*	Adjusted *R* ^2^	SEE
Total sample (*n* = 125)				
Age (years)	0.448	<0.01	0.250	1.423
Systolic blood pressure (mmHg)	0.357	<0.01		
Early postmenopause (*n* = 58)				
Systolic blood pressure (mmHg)	0.451	<0.01	0.252	1.363
Time in menopause (years)	0.249	0.04		
+1a (*n* = 17)				
Visceral fat level (points)	0.513	0.01	0.505	1.423
Diastolic blood pressure (mmHg)	0.445	0.03		
+1b (*n* = 15)				
Systolic blood pressure (mmHg)	0.639	0.01	0.362	0.522
+1c (*n* = 26)				
Systolic blood pressure (mmHg)	0.525	<0.01	0.348	0.284
Time in menopause (years)	0.369	0.03		
Late postmenopause (*n* = 67)				
Systolic blood pressure (mmHg)	0.274	0.02	0.158	1.505
Age (years)	0.274	0.02		

*Note*: +1a, end of the 12‐month period of amenorrhea required to define that the final menstrual period has occurred; +1b, 2‐year period after menopause when vasomotor symptoms are most likely; +1c, period of stabilization of high FSH (follicle‐stimulating hormone) levels and low estradiol values that is estimated to last 3–6 years; *p*, statistical significance; β, standardized coefficient; Adjusted *R* ^2^, correlation coefficient adjusted for the number of regressors.

Abbreviation: SEE, standard error of estimation.

When examining the subset of women who completed the cardiorespiratory test (*n* = 96; Table 5), EP women exhibited superior CRF, with a mean VO_2max_ that was 0.7 mL·kg^−1^·min^−1^ higher than the LP women (*p* = 0.02). No statistically significant differences were observed in the other variables analyzed.

**TABLE 5:** Comparison of physiological and clinical variables between women in early and late postmenopause according to cardiorespiratory fitness sample.

Variables	Total sample	Early postmenopause	Late postmenopause	Difference	*p*	Effect size (d)
	Mean ± SD	Mean ± SD	Mean ± SD			
	(*n* = 96)	(*n* = 48)	(*n* = 48)	Mean ± SD		
Age (years)	57.20 ± 6.42	52.88 ± 4.65	61.52 ± 4.86	−8.64 ± 1.21	0.31	−1.54
Full‐term pregnancies (*n*)	1.95 ± 0.91	1.96 ± 0.90	1.94 ± 0.93	−0.02 ± 0.01	0.80	−0.09
Duration of estrogen exposure (years)	37.46 ± 3.97	37.81 ± 4.16	37.11 ± 3.77	0.70 ± 0.39	0.54	−0.98
Weight (kg)	71.46 ± 11.86	74.56 ± 12.37	68.35 ± 10.56	6.21 ± 1.72	0.80	−1.13
Height (m)	1.58 ± 0.06	1.59 ± 0.05	1.57 ± 0.07	0.02 ± 0.01	0.07	−0.04
Fat mass (%)	40.42 ± 6.84	41.32 ± 6.53	39.53 ± 7.10	1.79 ± 0.57	0.53	1.52
Visceral fat level (points)	14.15 ± 4.40	15.02 ± 4.33	13.27 ± 4.34	1.75 ± 0.42	0.63	0.78
Systolic blood pressure (mmHg)	129.47 ± 14.68	126.19 ± 15.66	132.75 ± 12.98	−6.56 ± 2.72	0.18	9.37
Diastolic blood pressure (mmHg)	84.37 ± 7.98	86.02 ± 8.36	82.71 ± 7.31	3.31 ± 1.26	0.23	3.91
VO_2max_ (mL.kg^−1^.min^−1^)	24.73 ± 5.47	25.08 ± 5.03	24.38 ± 5.91	0.70 ± 1.09	0.02* ^,^ ^a^	−0.89

*Note*: *p*, statistical significance; Effect size (d), standardized difference between the means of the subgroups, measured using Cohen's d.

Abbreviations: SD, standard deviation; VO_2max_, maximal oxygen consumption.

^a^ Statistical analysis was performed using the ANCOVA test, controlling for age and blood pressure.

* *p* < 0.05.

Table 6 presents the stepwise regression models developed for CRF. The increase in % of fat mass was associated (*p* < 0.01) with a reduction in VO_2máx_ in the EP group (*β* = −0.589), and specifically at the stage +1c (*β* = −0.554). Among women who had been in estrogen depletion for more than 6 years, as well as those in stage +1a, increased central adiposity was associated with a reduction in aerobic fitness (*β* = −0.446; *p* < 0.01 and *β* = −0.364; *p* = 0.03, respectively). However, in stage +1a, this relationship was not independent of hormone therapy use, as indicated by the model, with an Adjusted *R* ^2^ of 0.824 (SEE = 2.107 mL·kg^−1^·min^−1^).

**TABLE 6:** Multiple regression analyses of cardiorespiratory fitness at distinct stages of menopause.

Maximum oxygen consumption	*β*	*p*	Adjusted *R* ^2^	SEE
Early postmenopause (*n* = 48)				
Body fat (%)	−0.589	<0.01	0.333	4.109
+1a (*n* = 13)				
Hormone therapy	0.686	<0.01	0.824	2.107
Visceral fat level (points)	−0.364	0.03		
+1c (*n* = 21)				
Body fat (%)	−0.554	<0.01	0.271	4.002
Late postmenopause (*n* = 48)				
Visceral fat level (points)	−0.446	<0.01	0.182	5.341

*Note*: +1a, end of the 12‐month period of amenorrhea required to define that the final menstrual period has occurred; +1c; period of stabilization of high FSH (follicle‐stimulating hormone) levels and low estradiol values that is estimated to last 3–6 years; *p*, statistical significance; β, standardized coefficient; Adjusted *R* ^2^, correlation coefficient adjusted for the number of regressors. Substage +1b was not included due to nonsignificant regression results. See Table S1.

Abbreviation: SEE, standard error of estimation.

## 4 DISCUSSION

The aim of this study was to describe how age, body composition, blood pressure, and menopausal characteristics relate to AS and CRF across early versus late postmenopause, and within the substages of early postmenopause stage. In this context, variables such as parity and the use of BP‐lowering medication did not show significant associations with AS or CRF.

The results revealed that SBP was a significant factor related to AS among postmenopausal women. Age and SBP together explained 25% of the variance in AS across the overall sample, underscoring the relevance of blood pressure management in this population. Within early postmenopause, longer time since menopause was associated with higher cfPWV (most notably in the +1c substage) while no such association was detected in the LP group, which is consistent with a potential plateau in vascular remodeling following prolonged estrogen deficiency. In the +1a EP substage, DBP and VFL together explained more than half of the variation in AS.

The measurement of AS, as indicated by cfPWV, revealed lower values in the EP group as shown by El Khoudary et al. (2020). This finding reflects a progressive increase in large‐artery stiffness across postmenopausal stages, in line with evidence that menopause is associated with reduced arterial elasticity and increased vascular remodeling (El Khoudary et al., 2020; Samargandy et al., 2020). Importantly, this comparison reflects relative differences within postmenopause; despite lower cfPWV in EP compared to LP, EP is still characterized by vascular changes when contrasted with premenopausal populations. Because this study did not include pre‐ or perimenopausal controls, inferences are restricted to within‐postmenopausal comparisons.

Among the 125 women included, the LP group was significantly older and exhibited higher SBP compared with the EP group. These factors tend to coexist in the LP and are associated with greater AS, consistent with prior reports in postmenopausal women (Samargandy et al., 2020; Tsai et al., 2017). Given the strong correlation between menopausal stage and age, the application of ANCOVA was not valid, and findings should be interpreted descriptively rather than as evidence of stage‐independent effects. Moreover, SBP emerged as a significant factor in nearly all substages, except for substage +1a. In the +1b substage, SBP was the only variable significantly associated with AS, accounting for 36.2% of the variance (Kodoth et al., 2022). Furthermore, SBP also remained significant in LP, further confirming its consistent influence on AS (Samargandy et al., 2022).

In EP, particularly in the +1c substage, longer time since menopause and elevated SBP were the primary factors associated with increased AS. This pattern is consistent with the progressive loss of estrogen's protective vascular effects, which normally help maintain arterial compliance (Davezac et al., 2021; Niță et al., 2021; Tap et al., 2020). In contrast, in LP women, prolonged estrogen deficiency may lead to a stabilization or “plateau” in arterial remodeling, which helps explain the absence of a significant association between time since menopause and cfPWV in that group (Niță et al., 2021; Raj et al., 2023). Elevated SBP further contributes to mechanical stress on the arterial wall, promoting structural changes and increased stiffness (Nowak et al. 2018).

In the first year following menopause (substage +1a), increased visceral fat and elevated DBP explained more than 50% of the variation in AS. Visceral adiposity contributes to metabolic dysfunction, inflammation, and endothelial impairment (Raj et al., 2023). Visceral fat secretes pro‐inflammatory cytokines (Opoku et al., 2023; Samargandy et al., 2020), reduces nitric oxide bioavailability, and impairs vasodilation (Nowak et al., 2018). Additionally, elevated DBP places constant stress on arterial walls, promoting remodeling and arterial calcification (Gersh et al., 2024; Lacolley et al., 2017). Combined, these factors may be particularly impactful during the early postmenopause, when the body is adjusting to rapid hormonal decline.

Results reveal that CRF was slightly lower in LP women compared to EP women, consistent with previous literature (Rael et al., 2021). No statistically significant differences were found between EP and LP in total BF or VFL. These findings align with literature evidence that although aerobic capacity declines with advancing postmenopause (Hulteen et al., 2022; Serviente et al., 2022), adiposity often increases during the initial years after menopause and then stabilizes (Marlatt et al., 2022). In our sample, higher BF percentage and VFL were inversely associated with CRF, reinforcing the established link between adiposity and lower CRF in postmenopausal women (Lesser et al., 2015; Lynch et al., 2002; Morardpour et al., 2020; Moreira et al., 2014). Consistent with evidence, total fat mass typically increases during the first 2 years after the final menstrual period and then stabilizes, which may help explain the absence of large between‐group differences in adiposity despite the lower CRF in LP (El Khoudary et al., 2020; Marlatt et al., 2022).

Across the sample, higher BF percentage in EP women, particularly in the +1c substage, is significantly associated with a reduction in CRF. Women in this stage exhibit elevated body fat levels, which are negatively linked with aerobic capacity (Lesser et al., 2015; Moreira et al., 2014), as shown by the strong inverse correlation between BF and VO_2max_ (Lesser et al., 2015; Lynch et al., 2002). This relationship suggests that increased adiposity during EP (El Khoudary et al., 2020; Fenton, 2021), especially when hormone levels stabilize (Pu et al., 2017), weakens CRF (Morardpour et al., 2020). It is important to note that the steepest decline in estrogen occurs in the 12 months preceding and following the final menstrual period, a phase characterized by substantial hormonal instability. After this rapid decrease, approximately 2–3 years into the postmenopausal period, estradiol levels stabilize at persistently low concentrations, a pattern that defines the +1c substage. These results are coherent with literature, which demonstrates that higher body fat in postmenopausal women contributes to decreased aerobic performance due to altered metabolism and cardiovascular function (Haapala, 2020; Lesser et al., 2015; Morardpour et al., 2020; Moreira et al., 2014).

Additionally, in stage +1a, VFL and hormone therapy explained over 82% of the variation in CRF, consistent with prior reports linking greater adiposity to reduced CRF (Granados et al., 2019; Lesser et al., 2015; Lynch et al., 2002; Moreira et al., 2014), which promotes fat accumulation in the abdominal region (Fenton, 2021). Also, visceral fat is linked to metabolic dysfunction and inflammation (Chomiuk et al., 2024; Larsen & Jansen, 2021), all of which impair cardiovascular function (Cesaro et al., 2023; Farb & Gokce, 2015) and reduce the efficiency of oxygen uptake (Lesser et al., 2015; Lynch et al., 2002), thereby lowering CRF. Conversely, hormone therapy is positively linked with VO_2máx_ in this stage, supporting the understanding that estrogen replacement helps mitigate the decline in aerobic capacity (den Ruijter & Kararigas, 2022; Matthews et al., 2018). Hormone therapy appears to help preserve VO_2máx_ in postmenopausal women by counteracting several physiological changes driven by estrogen loss. Estrogen plays a critical role in regulating fat distribution, maintaining muscle mass, mitochondrial efficiency, endothelial function, and cardio‐circulatory responsiveness, all of which are essential for sustaining aerobic fitness (Craighead et al., 2019; Morardpour et al., 2020). Literature corroborates these findings, as studies have shown that increased visceral fat is associated with reduced cardiorespiratory performance in postmenopausal women (Granados et al., 2019; Lee & Arslanian, 2019; Lesser et al., 2015; Moreira et al., 2014). Additionally, hormone therapy has been demonstrated to improve muscle function and reduce fat accumulation, preserving VO_2máx_ during the early postmenopausal period (Costa et al., 2020; Geraci et al., 2021; Tiidus et al., 2013).

Finally, in LP women, central adiposity was identified as an independent variable of CRF, with higher VFL significantly associated with lower CRF. Time since menopause was associated with CRF in our cohort, being higher in EP than in LP indicating a between‐stage difference within postmenopause. The pattern is consistent with body‐composition and hemodynamic correlates observed in our models. In LP, VFL was independently and inversely associated with CRF, whereas in +1a HT (positive) and VF (negative) jointly explained substantial variance. This supports a multifactorial interpretation, with central adiposity (and, in EP, HT exposure) more closely linked to CRF (El Khoudary et al., 2020; Fenton, 2021) in the first years post‐menopause (Costa et al., 2020; Geraci et al., 2021; Tiidus et al., 2013). As stated before, visceral fat contributes to metabolic dysfunction (Farb & Gokce, 2015), reducing the body's ability to transport and utilize oxygen efficiently (Larsen & Jansen, 2021).

Moreover, parity, particularly having multiple full‐term pregnancies, showed no association with AS or CRF in our cohort, neither across EP subgroups (*F* = 0.83, *p* = 0.44; Table 3). Notably, 73% of participants had 1–2 children, which may have limited the ability to detect effects that other cohorts have reported predominantly at higher parity (≥3), particularly for body composition phenotypes (e.g., greater adiposity) that could, in turn, relate to lower CRF (He et al., 2023; Kim et al., 2016; Morardpour et al., 2020; Zoet et al., 2019). Accordingly, our null findings should be interpreted in the context of this restricted parity range (Kim et al., 2016).

A key strength of this study is the careful selection of participants, with all individuals being postmenopausal women whose inclusion criteria were clinically verified and the standardized assessment of cfPWV and CRF across early and late postmenopause. This approach increases the reliability of group classification and minimizes potential classification bias. By considering different postmenopause stages, the study provided a structured framework for analyzing associations within this population.

The inclusion of different postmenopausal stages provided the study with a more robust methodological approach, sensitive to the heterogeneity of this population. Hormonal fluctuations, changes in body composition, and hemodynamic parameters occurring throughout reproductive aging distinctly influence CRF and AS, reinforcing the need for a segmented analysis. The integration of these variables enabled the identification of specific risk profiles and guided the development of more precise and effective intervention strategies, which are essential for promoting cardiovascular health and preserving quality of life in women after menopause.

Nonetheless, some constraints should be considered. The cross‐sectional design limits causal inference, and sample sizes within EP substages were modest. The strong correlation between age and menopausal stage excluded valid ANCOVA adjustments for SBP and DBP. In addition, bioelectrical impedance represents a doubly indirect method for assessing body composition. Although the equipment used features eight electrodes, the frequency spectrum employed does not exceed 100 kHz. Finally, the low prevalence of cfPWV >10 m/s and the relatively low use of hormone therapy further limited the power to detect smaller effects. These considerations do not detract from the observed patterns but highlight the need for longitudinal studies with larger samples and more precise gold‐standard phenotyping methods.

## 5 FUTURE PERSPECTIVES

It is recommended that future research prioritize longitudinal studies to elucidate causal relationships and assess the long‐term associations of lifestyle on cardiovascular health and physical fitness in postmenopausal women. A longitudinal design will facilitate a deeper understanding of how changes over time affect AS and CRF. This approach will increase our ability to assess the relationship of temporal changes on these variables, offering valuable insights for developing effective strategies to promote cardiovascular health within this population.

To complement this, employing more rigorous methodologies for assessing body composition, such as dual‐energy X‐ray absorptiometry, would provide more detailed and accurate information of body composition, allowing for improved discrimination between different body mass compartments. This would help identify specific alterations that could link to cardiovascular health.

Furthermore, investigating the relationship of natural versus induced menopause is essential to clarify how these distinct menopause experiences affect cardiovascular outcomes. This area of research is crucial, as the current literature remains insufficient in addressing the differences between these two menopause types and their relationship with cardiovascular health.

Also, it's important to focus on the effects of exercise amount on cardiovascular and overall health outcomes in postmenopausal women, specifically examining the differences between women with a lifelong history of regular physical activity and those who were previously inactive but began exercising later in life.

Further research in these areas will facilitate the improvement of intervention strategies and the upgrading of health outcomes for postmenopausal women.

## 6 CONCLUSIONS

In this postmenopausal cohort, AS (cfPWV) and SBP were lower in EP than in LP, indicating a relative within‐postmenopause difference rather than a vascular advantage versus perimenopause. Within EP, higher BF percentage was inversely associated with CRF, and VFL showed a consistent inverse association with CRF across EP and LP, with a stronger relationship in LP. In the +1a substage, hormone therapy was positively associated with CRF in models that also included visceral fat, although these exploratory findings warrant confirmation in larger samples.

Taken together, these results support stage‐targeted strategies that prioritize BP management, adiposity reduction, and exercise‐based interventions to preserve cardiovascular function and fitness as women transition from EP to LP. While our cross‐sectional design precludes causal inference, the observed patterns help refine hypotheses for longitudinal trials to test whether optimizing BP and body composition can attenuate the progression of vascular stiffening and decline in CRF during postmenopause.

## FUNDING INFORMATION

The author(s) disclosed receipt of the following financial support for the research, authorship, and/or publication of this article: This work was supported by National Funds by FCT—Portuguese Foundation for Science and Technology, under the projects: UID/04033/2025: Centre for the Research and Technology of Agro‐Environmental and Biological Sciences and LA/P/0126/2020 (https://doi.org/10.54499/LA/P/0126/2020); UID/04045: Research Center in Sports Sciences, Health Sciences, and Human Development; CIAFEL: (UIDB/00617/2020: doi: https://doi.org/10.54499/UIDB/00617/2020 and UIDP/00617/2020: doi: https://doi.org/10.54499/UIDP/00617/2020).

## CONFLICT OF INTEREST STATEMENT

The authors declare no conflicts of interest related to the content of this manuscript.

## ETHICS STATEMENT

This research complied with institutional ethical standards and the Declaration of Helsinki. All participants gave written informed consent before any study procedures. The protocol received prior approval from the Ethics Committee of the University of Trás ‐os ‐Montes and Alto Douro (approval Doc108 ‐CE ‐UTAD ‐2022). Data were handled in anonymized form, and no identifiable information or participant images are presented.

## Supporting information


Table S1.

## Data Availability

The datasets generated and analyzed during the current study are available from the corresponding author on reasonable request.
