# Supplementary material for: Exploring the relationship between reproductive aging, arterial stiffness, and cardiorespiratory fitness in women after menopause
Source: Physiol Rep. 2026 Mar 29;14(7):e70722. doi: 10.14814/phy2.70722 (PMC13140205; doi:10.14814/phy2.70722)
Supplement: Supplementary file 1 — Table S1. [file PHY2-14-e70722-s001.docx]

**Supplementary Table 1:** Non-significant predictors from multiple regression models of cardiorespiratory fitness.

| **Predictor Variable** | **β** | **p-value** | **Interpretation** |
| --- | --- | --- | --- |
| Body fat (%) | -0.220 | 0.112 | Not significant |
| Age (years) | -0.150 | 0.245 | Not significant |
| SBP (mmHg) | -0.105 | 0.312 | Not significant |

Model R² = 0.083, p = 0.214; Standard Error of Estimation (SEE) = 4.932; **SBP**; Systolic blood pressure.
